# Molecular Components and Functions of the Endocannabinoid System in Mouse Prefrontal Cortex

**DOI:** 10.1371/journal.pone.0000709

**Published:** 2007-08-08

**Authors:** Mathieu Lafourcade, Izaskun Elezgarai, Susana Mato, Yamina Bakiri, Pedro Grandes, Olivier J. Manzoni

**Affiliations:** 1 INSERM U862, Equipe Physiopathologie de la plasticité synaptique, Bordeaux, France; 2 Department of Neurosciences, Faculty of Medicine and Dentistry, University of the Basque Country, Bilbao, Spain; The Rockefeller University, United States of America

## Abstract

**Background:**

Cannabinoids have deleterious effects on prefrontal cortex (PFC)-mediated functions and multiple evidences link the endogenous cannabinoid (endocannabinoid) system, cannabis use and schizophrenia, a disease in which PFC functions are altered. Nonetheless, the molecular composition and the physiological functions of the endocannabinoid system in the PFC are unknown.

**Methodology/Principal Findings:**

Here, using electron microscopy we found that key proteins involved in endocannabinoid signaling are expressed in layers V/VI of the mouse prelimbic area of the PFC: presynaptic cannabinoid CB1 receptors (CB1R) faced postsynaptic mGluR5 while diacylglycerol lipase α (DGL-α), the enzyme generating the endocannabinoid 2-arachidonoyl-glycerol (2-AG) was expressed in the same dendritic processes as mGluR5. Activation of presynaptic CB1R strongly inhibited evoked excitatory post-synaptic currents. Prolonged synaptic stimulation at 10Hz induced a profound long-term depression (LTD) of layers V/VI excitatory inputs. The endocannabinoid -LTD was presynaptically expressed and depended on the activation of postsynaptic mGluR5, phospholipase C and a rise in postsynaptic Ca^2+^ as predicted from the localization of the different components of the endocannabinoid system. Blocking the degradation of 2-AG (with URB 602) but not of anandamide (with URB 597) converted subthreshold tetanus to LTD-inducing ones. Moreover, inhibiting the synthesis of 2-AG with Tetrahydrolipstatin, blocked endocannabinoid-mediated LTD. All together, our data show that 2-AG mediates LTD at these synapses.

**Conclusions/Significance:**

Our data show that the endocannabinoid -retrograde signaling plays a prominent role in long-term synaptic plasticity at the excitatory synapses of the PFC. Alterations of endocannabinoid -mediated synaptic plasticity may participate to the etiology of PFC-related pathologies.

## Introduction

The endogenous cannabinoid (endocannabinoid, eCB) system is emerging as one of the most ubiquitous activity dependent regulatory system in the CNS [1], [2]. The wide expression of cannabinoid CB1 receptors (CB1R) explains the ever growing list of functions attributed to the eCB-system (for extensive reviews see [1]–[3]. Thus, pharmacological agents acting on the various elements of the eCB system have a great potential to treat a wide range of pathologies including food intake disorders, chronic pain, emesis, insomnia, glaucoma, glioma, motor disorders, stroke and severe psychiatric conditions such as depression, autism and schizophrenia [2], [4]–[6].

Schizophrenia is a chronic and severe brain disease that has its symptomatic onset in early adulthood and affects multiple cognitive and behavioral functions. Prefrontal dopaminergic and glutamatergic dysfunctions have been proposed to participate to the etiology of schizophrenia [7]. Deregulations of the eCB system in the prefrontal cortex (PFC) may also participate to this disease [8]. The PFC participates to the organization and the planning of voluntary movements and to the programming of actions [9]. The PFC allows the storage of information and their subsequent use for decision taking and the elaboration of strategies. In particular, the PFC has been proposed to play a crucial role in short-term working memory (the ability to keep events “in mind” to prepare organized behavioral responses, [9]). Cannabis derivatives alter prefrontal functions such as working memory and a number of studies suggest that cannabis use can cause or exacerbate psychoses and may increase the risk of developing schizophrenia [10]–[12]. Furthermore, increased density of binding at CB1R in the PFC of schizophrenics has been demonstrated [13], [14] while other studies have shown increased anandamide levels in the CSF or blood of schizophrenics [15]–[17]. In addition genetic studies have shown an association between the gene encoding CB1R (CNR1) and schizophrenia [18], [19]. Finally, the genetic ablation of CB1R alters the schizophrenia-like behavioral effects of the dissociative anesthetic and non-competitive NMDAR antagonist phencyclidine [8], [20]. Thus multiple evidences point toward a role of the eCB-system in the pathophysiological functions of the PFC [8], [21]. Although the PFC appears as a structure of choice to study the eCB system, how CB1Rs play a role in synaptic transmission and plasticity within the PFC is poorly documented [22]–[24]. Here, we used electron microscopy and patch-clamp techniques to describe the molecular components of the eCB system and how they participate to long-term synaptic plasticity at pyramidal synapses in layers V/VI synapses of the mice prelimbic area of the PFC.

## Results

### Ultrastructural localization of components of the eCB system in mice prefrontal cortex pyramidal cells

Confocal and electron microscopy approaches were used to identify the localization of proteins known to participate to eCB-mediated retrograde signaling in the striatum and the hippocampus [1], [25], [26], [27], [28], [29]. Thus in layers V/VI synapses of the prelimbic area of the prefrontal cortex (PrPFC), we searched for the presence of: CB1R, mGluR5, a postsynaptic receptor to glutamate which activation releases eCB [28] and DAG lipase 1α (DGL-α ), an enzyme that produces 2-AG.

**Figure 1:**
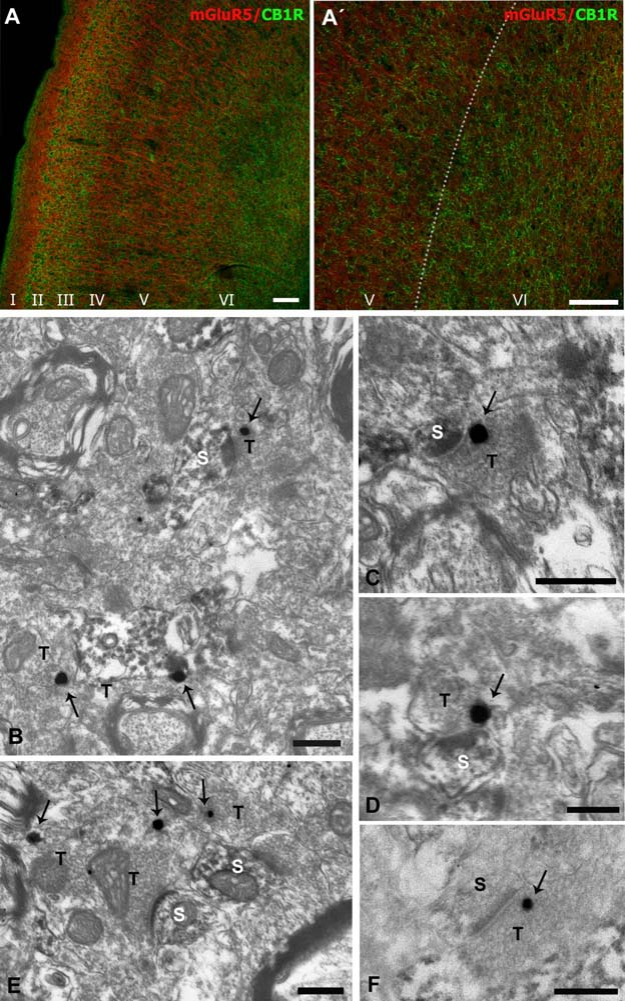
Immunocytochemical localization of mGluR5 and CB1R in the prelimbic prefrontal cortex (prPFC). (A) Double confocal immunofluorescence showed no colocalization of both proteins. mGluR5 immunoreactivity was distributed in the neuropil throughout cortical layers, but the staining was more evident in apical dendrites of layers V/VI pyramidal neurons heading for the superficial layers. High CB1R immunoreactivity was in layers II/III, deeper part of layer V and throughout layer VI (Á: enlargement of A). The lack of CB1R labeling showed mGluR5 immunoreactivity in layers I, IV and in the upper part of layer V. (B–F). Electron microscopy of the localization of mGluR5 (DAB immunoreaction product) and CB1R (silver-intensified gold particles) in prPFC cortical layers V/VI. mGluR5 immunoreactivity was in dendritic profiles and spines (s). Typically, CB1R immunopositive synaptic terminals (T) made asymmetric synapses with mGluR5-immunoreactive dendritic spines (s). Note metal particles localizing CB1R (arrows) at perisynaptic and extrasynaptic sites relative to presynaptic membrane specializations of axonal synaptic boutons. Scale bars in A,A': 100 µm; B,C,E,F: 0.33 µm; D:0.20 µm.

Double immunofluorescence for mGluR5 and CB1R showed no colocalization of both proteins (Fig 1A, A'). Thus, mGluR5 labeling was in cell bodies and ascending dendritic bundles of deep cortical pyramidal neurons (Fig 1A). Furthermore, a mesh-like immunoreactivity was also observed in the neuropil throughout the prefrontal cortical layers (Fig 1A). The CB1R expression pattern was layer specific, as immunofluorescence was mostly restricted to layers II/III and V/VI (Fig 1A, A'). Immunopositive dotty elements and thin processes took up the neuropil of the labeled layers where they intermingled with mGluR5 immunoreactive somatodendritic domains (Fig 1A, A').

The precise localization of CB1R and mGluR5 in cortical layers V/VI was studied by double immunoelectron microscopy (Fig 1B–F). As expected, mGluR5 immunoreactivity was in small dendritic spines. Although DAB immunodeposits diffused within the profiles, postsynaptic densities of asymmetrical synapses were observed in mGluR5 positive spines receiving CB1R immunolabeled synaptic terminals. Furthermore, the CB1R silver-intensified gold particles were closely placed to membranes away from presynaptic specializations of the axon terminals.

**Figure 2:**
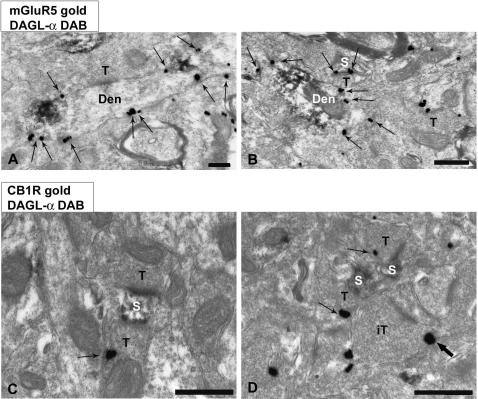
Immunocytochemical localization of mGluR5/DGL-α (A, B) and CB1R/ DGL-α (C, D) in mouse prPFC cortical layers V/VI. Double preembedding immunogold and immunoperoxidase methods for electron microscopy. (A, B) mGluR5 metal particles (arrows) and DGL-α immunodeposits colocalized in postsynaptic dendrites (den) and dendritic spines (s). mGluR5 labeling was in perisynaptic and extrasynaptic membranes. No mGluR5/DGL-α immunoreactivity was observed in presynaptic terminals (T). (C, D) CB1R immunoparticles were on presynaptic terminals membranes (T) away from synaptic specializations made on postsynaptic DGL-α-immunoreactive dendritic spines (s). Observe that DGL-α-positive spines also received CB1R-immunonegative synaptic terminals, and that a CB1R-labeled presynaptic terminal (thick arrow) probably of inhibitory nature (IT in D) made a synapse with a postsynaptic DGL-α-negative dendritic branchlet. Scale bars: 0.5 µm.

To define the subcellular distribution of DGL-α with respect to CB1R and mGluR5, we performed double immunoelectron microscopy by immunoperoxidase for DGL-α and by a preembedding immunogold method for either mGluR5 or CB1R (Fig 2A–D). Indeed, DGL-α immunoreactivity colocalized with mGluR5 in dendritic and spiny domains (Fig 2A, B). As expected, mGluR5 immunoparticles were perisynaptic or far excluded from postsynaptic specializations. Conversely, CB1R immunolabeling was on membranes of axon terminals making synapses with DGL-α immunoreactive dendritic spines (Fig 2C, D). CB1R immunoparticles were left out of the presynaptic specializations of the synapses. Quantified data are summarized Table 1.

**Table 1:** Percentage of presynaptic and postsynaptic elements immunoreactive for CB1R, mGluR5 or DGL-α.

Protein	Profile	% of immuno+profiles	Particles/µm±SEM	mGluR5+ (DAB) Postsynaptic	DGL-α	Total n° of profiles	Analyzed area (µm^2^)
CB1R	Presynaptic (terminals)	51.28%	1.167±0.132	87%	63.6%	80+/76-	339.66
mGluR5	Postsynaptic (den-spines)	79.88%	2.015±0.11	----------	55.1%	135+/34-	257.55
DGL-α	Postsynaptic (den-spines)	55.84%	1.286±0.16	99%	--------	86+/68-	305.92

The percentage of CB1R-labeled presynaptic compartments relative to postsynaptic mGluR5 and DGL-α is shown. Prelimbic prefrontal cortical sections used for counting were obtained from 3 adult mice. Electron micrographs were taken at a final magnification of x15,000.

### Cannabinoids acting at presynaptic CB1R inhibits glutamatergic transmission in the mice prefrontal cortex

The presence of CB1R immunolabeling at the axon terminals of (presumably glutamatergic) asymmetrical synapses prompted us to test at the functional level the effects of CB agonists at the glutamatergic synapses between layer 2/3 and layer 5/6 pyramidal cell layers of the mice PrPFC.

Whole cell voltage clamp recordings were performed to measure the effects of CB1R activation on excitatory synaptic responses evoked in the presence of the GABA-A antagonist picrotoxin (100 µM) by stimulating layer 2/3 to layer 5/6 pyramidal cells synapses. Noteworthy, to reduce as much as possible polysynaptic events [22], we used a physiological external Ca^2+^ concentration (1.2 mM) that in most cases prevented the occurrences of polysynaptic currents (not shown).

**Figure 3:**
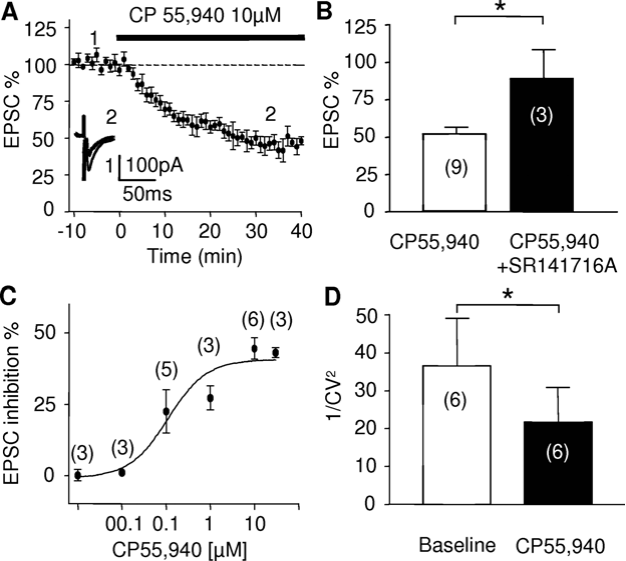
Pharmacological characterization of presynaptic CB1R at layer V-VI synapses of the PrPFC. Layer V-VI pyramidal cells were voltage-clamped and held at -70mV. (A) CB1R-mediated inhibition of evoked transmission. The cannabimimetic CP55,940 (10 µM) reduced evoked EPSCs on average to 48±5 % (n = 6) of basal value. Traces represent the average of 10 consecutive EPSCs taken at the times indicated on the time-course graph. (B) The inhibitory effects of CP55,940 on evoked EPSCs were blocked by pre-treatment with the selective CB1R antagonist SR141716A (10 µM, t-test p = 0.0386) in agreement with the involvement of CB1R. (C) Dose response curve measured 20 min after beginning CP55,940 application. Each point is expressed as the percentage of inhibition of its basal value. The EC50 was 195±0.3 nM. (D) The coefficient of variation, expressed as 1/CV^2^ was reduced following the CP55,940 (p = 0.0107 paired t-test). 1/CV^2^ was calculated with 60 sweeps i.e. 10 min before and 20 min after CP55,940.

Evoked excitatory postsynaptic currents (eEPSCs) in layer 5/6 were strongly inhibited by bath perfusion of the CB agonist CP 55,940 (10 µM, Fig 3A). The inhibitory effects of the CB agonist were totally prevented by pretreatment and co-perfusion with the selective CB1 antagonists SR141716A (10 µM, Fig 3B) demonstrating the implication of cannabinoid receptors of the CB1R subtype. The effects of the CB agonist CP55,940 were dose-dependent with an EC_50_ of 195±0.3 nM (Fig 3C), in agreement with other reports [30], [31]. Taken together, these data show that the inhibitory effects of the CB agonist are due to the activation of CB1 receptors.

To functionally assess the origin of the CB1-mediated depression, we measured the coefficient of variation (CV = standard deviation/mean amplitude) of individual evoked EPSCs. Bath application of CP55,940 (10 µM) significantly decreased the coefficient of variation, expressed as 1/CV^2^ (n = 6, Fig 3D). Together with our ultrastructural data, these electrophysiological results are consistent with a presynaptic locus of action of CB1R.

### eCB-mediated LTD in prefrontal cortex pyramidal cells

**Figure 4:**
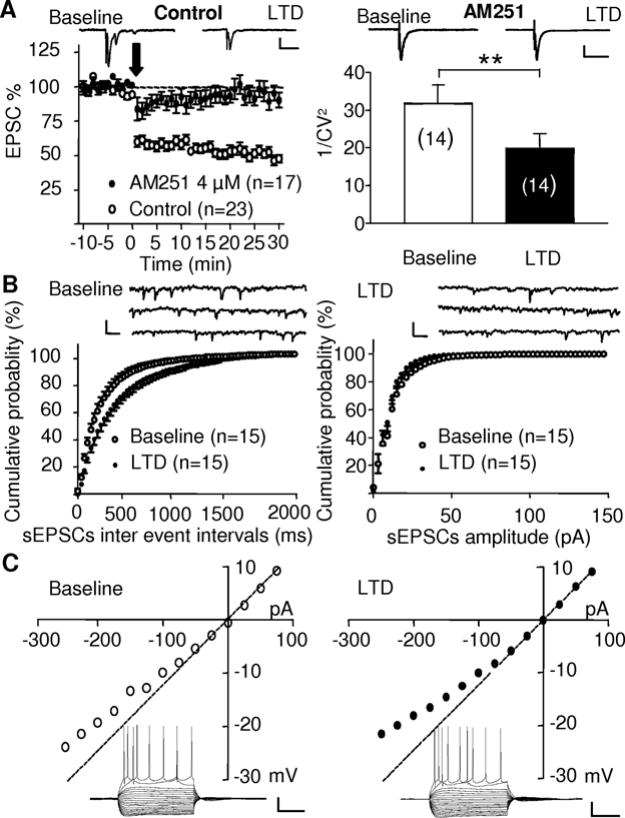
Presynaptic CB1R-mediated LTD in the PrPFC. (A) Left: A 10 min 10 Hz stimulation of layer II-III fibers (arrow) induced a profound long-term depression of evoked EPSCs recorded in patch-clamped layer V-VI pyramidal neurons. The induction of LTD was completely prevented when slices were preincubated and tetanized in the presence of the CB1R antagonist AM251 (4 µM). Traces represent the average of 12 consecutive EPSCs before and 25 minutes after LTD induction in the absence (upper left) or presence (upper right) of AM251. Right: The coefficient of variation, 1/CV^2^, was significantly reduced after induction of eCB-LTD (p = 0.0025 paired t-test). Calibration bars: x: 50 ms, y: 100 pA. (B) Representative continuous 3 seconds sweeps showing the spontaneous EPSCs (sEPSCs) recorded before (left) and after eCB-LTD induction (right). The distribution of sEPSCs inter-event intervals (left panel) but not of their amplitude (right panel) was modified following induction of LTD suggesting a presynaptic modulation (Kolmogorov-Smirnov test: inter event interval p<0.005, amplitude p = 0.507). Calibration bars: x: 100 ms, y: 10 pA. (C) Responses to hyperpolarizing and depolarizing somatic current pulses of a typical pyramidal neuron in the PFC before and after induction of eCB-LTD. Similar I–V curves were obtained suggesting that eCB-LTD induction did not change post-synaptic pyramidal neurons properties. Calibration bars: x: 100 ms, y: 25 mV.

**Figure 5:**
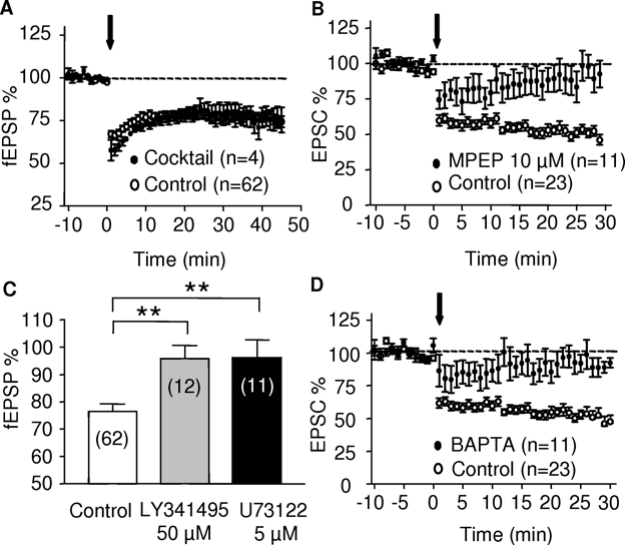
Postsynaptic receptors and transduction pathways involved in eCB-LTD. (A) eCB-LTD was not affected by a mixture of the NMDAR antagonist MK801 (40 µM), the D1 receptor antagonist SCH23390 (25 µM) and the D2 receptor antagonist sulpiride (25 µM). (B) The mGluR5 antagonist MPEP (10 µM) completely blocked eCB-LTD (C) Bar graph summarizing experiments showing that the non subtype selective group 1mGluR antagonist LY341495 (50 µM) and the Phospholipase C inhibitor U73122 both prevented eCB-LTD induction. 45 min after the end of the tetanus, the fEPSPs was 76.6±2.64% (n = 62) of baseline in control and 95.8±4.85% (n = 12, p = 0.004 t-test) and 96.33±6.4% (n = 11, p = 0.0009 t-test) in LY341495, respectively. (D) eCB-LTD requires postsynaptic Ca^2+^ rise. Time course of all the experiments performed where the recording pipette was filled with BAPTA (20 mM, n = 11) and where eCB-LTD was completely blocked.

The eCB system mediates retrograde long-term synaptic plasticity at the PrPFC to nucleus accumbens glutamatergic synapses [28], [32]–[34] and in other brain areas [1]. Based on our present results showing functional presynaptic CB1R at pyramidal cells synapses and our ultrastructural data showing the presynaptic localization of CB1R, we tested the existence of eCB-dependent plasticity within the PrPFC. We used a stimulation protocol that we first introduced [28] and that is based on naturally occurring frequencies [35], [36]. Stimulation of layer 2/3 PrPFC afferents to layer 5/6 pyramidal cells (10 min at 10 Hz) induced a robust LTD of glutamatergic inputs that can be reliably observed when recording pyramidal cells in the whole cell patch-clamp configuration (Fig 4A left), as well as when recording extracellular field excitatory post synaptic potentials (fEPSPs, Fig 5A). This LTD was mediated by eCBs/CB1R since it was completely prevented when slices were preincubated with the CB1R antagonists AM251 (Fig 4A left). The eCB-LTD was expressed at a presynaptic locus: first, the coefficient of variation, expressed as 1/CV^2^, was reduced following LTD induction (Fig 4A right) and second, the frequency but not the amplitude of spontaneous EPSCs (sEPSCs) was reduced following LTD induction (Fig 4B). Finally, we observed that the basal membrane properties and firing pattern of pyramidal cells were not affected following induction of LTD (Fig 4C).

### eCB-LTD is mediated by postsynaptic mGluR5

In the PrPFC, eCB-LTD did not require the activation of NMDAR, D1 or D2 dopamine receptors, as demonstrated by the lack of effects of a cocktail of antagonist of these receptors (Fig 5A). Postsynaptic metabotropic receptors (mGluRs) of the mGluR5 subtype translate glutamatergic signaling into eCB retrograde signaling [28], [32]. In the PrPFC synapses, we found that both the non-subtype selective mGluR antagonist LY341495 (50 µM, Fig 5C grey bar) and the specific mGluR5 non-competitive antagonist MPEP (10 µM, Fig 5B) blocked eCB-LTD. Thus, as previously described at the PrPFC to nucleus accumbens synapses [28], activation of mGluR5 is necessary to induce eCB-LTD via a yet unidentified eCB that activates presynaptic CB1R. This observation underscores the importance of mGluR5 in eCB-mediated phenomena throughout the brain [1].

### Phospholipase C and postsynaptic Ca^2+ ^rise are necessary for eCB-LTD

Phospholipase C (PLC) is a primary intracellular effector of both mGluR1 and mGluR5 group 1 mGluRs [37] and we tested the effects of the Phospholipase C inhibitor U73122 on eCB-LTD. As summarized figure 5C (black bar) U73122 (5 µM) completely blocked eCB-LTD. Activation of PLC-coupled mGluR results in IP3-mediated release of Ca^2+^ by intracellular stores [37]. Accordingly, we found that chelation of postsynaptic Ca^2+^ (with 20 mM BAPTA intrapipette) completely prevented the induction of eCB-LTD at PrPFC synapses (Fig 5D), as in the nucleus accumbens synapses [28]. These results strongly indicate that the intracellular cascade triggered by the stimulation of mGluR5 involves the PLC and that postsynaptic Ca^2+^ rise is required to induce eCB-LTD, in accord with a role for postsynaptic mGluR5.

### 2-AG mediates eCB-dependent LTD in the prefrontal cortex

**Figure 6:**
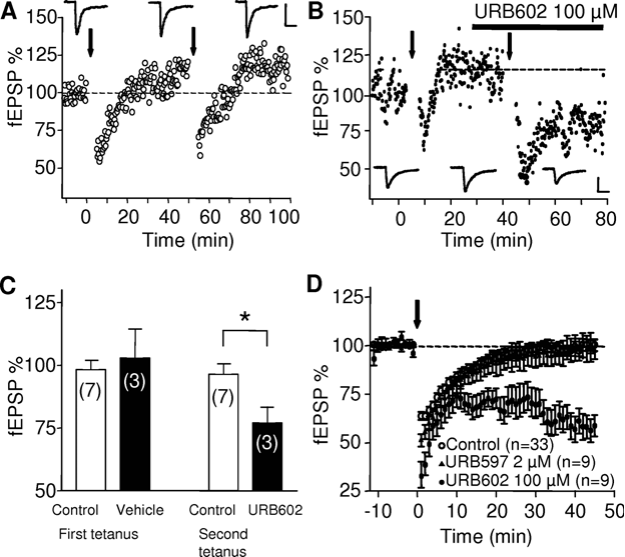
Role of 2-AG in eCB-LTD LTD in the PrPFC. (A) Typical experiment showing that a 5 min stimulation at 10 Hz is sub threshold to induce LTD, even when applied two consecutives times. Calibration bars: x: 10 ms, y: 0.2 mV. (B) Representative experiment showing that 5 min at 10 Hz can induce LTD when applied after bath perfusion with URB 602 (100 µM). Traces were taken at the time indicated on corresponding graph. Calibration bars: x: 10 ms, y: 0.2 mV. (C) Summary bar histogram of all the experiments performed where the first tetanus was given in saline and the second tetanus was given after bath perfusion of URB602. LTD was induced only when URB was present (t-test, p = 0.0271). (D) Averaged time courses of the experiments in which the 5min at 10Hz protocol was given in control ACSF (open circles) of after pre-treatment with URB597 (2 µM, black triangles) or URB602 (black circles).

**Figure 7:**
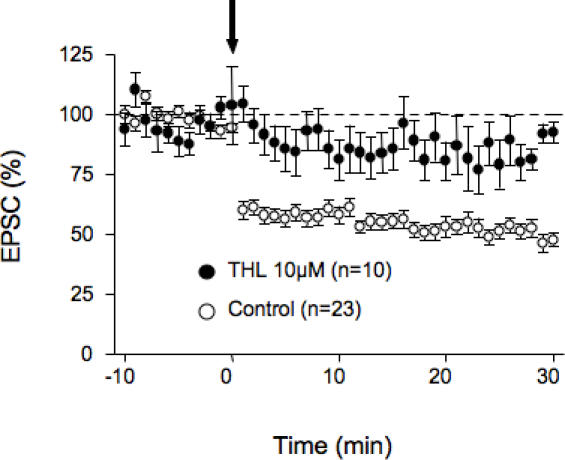
Inhibition of DGL-α, the enzyme that synthesizes 2-AG, blocked eCB-LTD. Averaged time courses of the experiments in which the 10 min at 10 Hz protocol was given in control ACSF (open circles) of after pre-treatment with tetrahydrolipstatin (THL, 10 µM, black circles), an inhibitor of the DGL-α.

**Figure 8:**
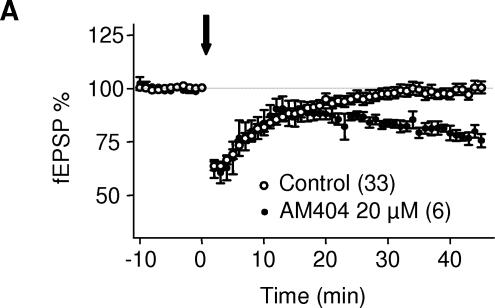
Effects of blocking eCB reuptake on sub threshold tetanus. Averaged time courses of the experiments in which the 5 min at 10 Hz protocol was given in control ACSF (open circles) of after pre-treatment with the eCB reuptake blocker AM404 (20 µM, black circles).

The two main eCBs produced upon neuronal stimulation in the brain are 2-AG and anandamide [38]. In an attempt to identify the molecular nature of the eCB responsible for LTD in the PrPFC, we used a stimulation protocol (5 min at 10 Hz) that was normally sub threshold to induce LTD in our preparation (Fig 6A,D). We next took advantage of the recent development of specific inhibitors of the enzyme degrading 2-AG, Monoacyl Glycerol lipase [39], [40]. When PrPFC slices were perfused with the specific Monoacyl Glycerol lipase inhibitor URB602 (100 µM) [39], [40], the “5 min at 10Hz” protocol induced LTD showing that 2-AG degradation limits the induction of LTD (Fig 6B-C). In contrast, blockade of the Fatty Acid Amide Hydrolase, the enzyme that breaks anandamide down, with the specific inhibitor URB597 (2 µM) had no effect (Fig 6D). Importantly, we observed that blocking DGL-α with tetrahydrolipstatin (THL, 10 µM, 2 hours preincubation) strongly inhibited eCB-LTD (Figure 7). All together these experiments strongly suggest that 2-AG and not anandamide mediates eCB-LTD in the PrPFC. Finally, in slices pretreated with AM404 (20 µM), an inhibitor of eCB uptake, the “5 min at 10 Hz” protocol was sufficient to induce LTD showing that eCB uptake is an important limiting step in the induction of 2-AG-mediated plasticity (Fig 8).

## Discussion

The present study reports several novel findings regarding the eCB system in the mice PFC. First, we identified several components of the eCB system at the synapses on layer V/VI pyramidal neurons: CB1R are located on axon terminals that make contacts with postsynaptic densities bearing both mGluR5 - a metabotropic glutamate receptor known for its central role in coupling glutamate release to eCB-production [1], [28], [32], [41], [42] - and DGL-α , a key enzyme of the synthesis of 2-AG. Second, in agreement with the molecular composition of the eCB system at these PFC synapses, we discovered that the eCB-system mediates a LTD of evoked excitatory responses.

The description of the localization of the molecular components of the eCB-system is a necessary step toward the understanding of the physiological roles of the eCB system. In a recent study, Katona and colleagues [29] demonstrated for the first time the precise anatomical position of CB1R and of DGL-α at the excitatory synapse of the hippocampus: presynaptic CB1R are localized opposite to dendritic apparatus expressing DGL-α Our results confirm and extend those of Katona. In synapse of the PFC, CB1R and DGL-α are also expressed on opposite sides of –presumably glutamatergic synapses. In addition, we provide the first evidences that in the postsynaptic compartments, the DGL-α is localized closely to mGluR5, a glutamate receptor well known for its key role in eCB-retrograde plasticity. Thus, our data demonstrate that postsynaptic spines in the PFC contain the two key proteins necessary to translate glutamatergic signals (mGluR5) into eCB (DGL-α) [26].

Our electron micrographs also show that presynaptic CB1R face postsynaptic mGluR5 at PFC synapses. Together, these data reinforce the proposition that “this molecular composition of the eCB system may be a general feature of most glutamatergic synapses…” [29].

Stimulation of layers II/III and/or layers V/VI fibers at 10Hz induced a remarkably robust LTD of evoked and spontaneous EPSCs recorded in layers V/VI pyramidal neurons. Thus, as we first demonstrated at the PFC to accumbens glutamatergic synapses [28], prolonged stimulation at frequencies around 10Hz is optimal to induced eCB-mediated LTD [32]–[34], [43]–[45]. The reasons for the efficiency of this frequency range are not clear but it is possible that this frequency allows for the full activation of perisynaptic mGluR5 [46]. Noteworthy, similar frequencies have been recorded in-vivo in behaving rats [35], [47], [48], underlying the importance of using physiologically relevant stimuli when studying synaptic plasticity ex vivo.

The most abundant eCBs released following neuronal synaptic activity are 2-AG and anandamide [38]. In most brain areas, pharmacological experiments using blocker of eCB synthesis suggest that eCB-mediated synaptic plasticity involves 2-AG rather that anandamide [1], [39], [40], [49]–[55]. Noteworthy, anandamide but not 2-AG seems to play a predominant role in the regulation of synaptic plasticity in the striatum [56]. We found that it is possible to induce eCB-LTD in the PrPFC by applying subthreshold stimulation in the presence of blockers of either eCBs reuptake (AM404) or 2-AG degradation [39], [40]. In contrast, blocking anandamide degradation (using the FAAH inhibitor URB597) had no effect. Thus our data indicate that 2-AG degradation and eCB reuptake are important limiting steps in the induction of 2-AG-mediated synaptic plasticity. The prominent role of 2-AG at PrPFC synapses is supported by our findings that the enzyme producing 2-AG, the DGL-α and mGluR5 colocalize in postsynaptic specializations. Interestingly, activation of group 1 mGluRs-such as mGluR5- has been clearly linked to the production of 2-AG [42]. Finally we observed that blocking the DGL-α with THL, strongly inhibited eCB-LTD (Figure 7). We conclude that 2-AG mediates eCB-LTD in the PrPFC.

Based on the finding that cannabinoids potentiate emotional learning plasticity in the medial PFC, it has recently been proposed that the eCB system plays a major role in the neural circuits underlying emotional learning [21], [57]. Moreover, the PFC plays a central role in adaptive behaviors such as the extinction of aversive memories [58], [59], which is one of the best documented physiological functions mediated by the eCB system [60], [61]. Thus, our data reinforces the idea that the eCB-system is a critical component of PFC physiology and that deregulation of eCB-mediated synaptic plasticity may participate to the etiology of PFC-related pathologies.

## Materials and Methods

### Animal treatment

Animal experiments accomplished the criteria of the European Communities Council Directive (86/609/EEC) and the United States National Institutes of Health *Guide for the Care and Use of Laboratory Animals*. Mice (4–6 weeks old male, C57B1/6 strain) were housed, grouped and acclimatized to laboratory conditions (12 hr light/dark cycles) 1 week before the experiment and had *ad libitum* food and water access.

### Slice preparation and electrophysiology

Whole cell patch-clamp and extracellular field recordings were made from visualized pyramidal cells in coronal slices of mouse prefrontal cortex. In brief, mouse were anesthetized with isoflurane and decapitated. The brain was sliced (300 µm) in the coronal plane using a vibratome (Integraslice, Campden Instruments, Loughborough, UK) and maintained in physiological saline at 4°C. Immediately after cutting, slices containing the PrPFC were stored for 30 min at 32–35°C in artificial cerebrospinal fluid (ACSF) that contained (in mM): 126 NaCl, 2.5 KCl, 2.4 MgCl_2_, 1.2 CaCl_2_, 18 NaHCO_3_, 1.2 NaH_2_PO_4_, and 11 Glucose, and was equilibrated with 95% O_2_/5% CO_2. _Slices were stored at room temperature until time of recording. For recording, slices were placed in the recording chamber and superfused (2 ml/min) with ACSF. All experiments were done at 32–35°C. The superfusion medium contained picrotoxin (100 µM) to block GABA_A_ receptors. All drugs were added at the final concentration to the superfusion medium.

To evoke synaptic currents, stimuli (100–150 µsec duration) were delivered at 0.1 Hz through a glass electrode filled with ACSF and placed either in layer 2/3 or in layer 5/6 as previously described [22]. No difference was observed between the two sites of stimulation and thus data were pooled together. Both the EPSC area and amplitude were measured (graphs depict amplitudes).

For extracellular field experiments, the recording pipette was filled with ACSF. Both the field excitatory postsynaptic potential (fEPSP) area and amplitude were measured (graphs depict area). The glutamatergic nature of the extracellular fEPSP was confirmed at the end of the experiments through the application of the non-NMDA ionotropic glutamate receptor antagonist DNQX (20 µM), which completely blocked the synaptic N2 component without altering the non-synaptic N1 component (not shown).

For whole cell patch-clamp experiments, layer 5/6 pyramidal neurons were visualized using an upright microscope with infrared illumination. Recordings were made with electrodes containing the following (mM): Cesium Methane-Sulfonate (CH_3_O_3_SCs) or K^+^Gluconate 128, NaCl 20, MgCl_2_1, EGTA 1, CaCl_2_ 0.3, Na^2+^-ATP 2, Na^+^-GTP 0.3, Glucose 10 buffered with Hepes 10, pH 7.3, osmolarity 290 mOsm. Electrode resistance was 4–6 MOhms. For the BAPTA experiments, 20 mM BAPTA was added to the intracellular medium.

A −2 mV hyperpolarizing pulse was applied before each evoked excitatory post-synaptic current (EPSC) in order to evaluate the access resistance, and those experiments in which this parameter changed >20% were rejected. Access resistance compensation was not used and acceptable access resistance was <25 MOhms. The potential reference of the amplifier was adjusted to zero prior to breaking into the cell. An Axopatch-1D (Molecular Device, Sunnyvale, USA) was used to record the data, which were filtered at 1–2 kHz, digitized at 5 kHz on a DigiData 1200 interface (Molecular Device, Sunnyvale, USA) and collected on a PC using Clampex 9.2 and analyzed using Clampfit 9.2 (Molecular Device, Sunnyvale, USA).

Spontaneous miniature excitatory postsynaptic currents (sEPSCs) were recorded in the whole cell voltage-clamp configuration using Axoscope 9.2 (Molecular Device, Sunnyvale, USA). sEPSC amplitude and inter-interval time were detected and analyzed using Clampfit 9.2 (Molecular Device, Sunnyvale, USA). For this analysis, a template of sEPSCs generated from averaging several typical synaptic events was slid along the data trace one point at a time. At each position, this template is optimally scaled and offset to fit the data and a detection criterion is calculated. The detection criterion is the template-scaling factor divided by the goodness-of-fit at each position. An event is detected when this criterion exceeds a threshold and reaches a sharp maximum.

The coefficient of variation (CV) was calculated for as standard deviation / mean amplitude of individual evoked EPSCs and expressed as 1/CV^2^.

### Data analysis and materials

All values are given as mean±S.E.M. For field recording/patch-clamp experiments, *n* corresponds to the number of individual cells/slices analyzed, with at least 4 animals included in each condition. Statistical significance between groups was tested using the Mann-Whitney U-test. Kolmogorov-Smirnov test was used for the statistical comparison of the cumulative distributions. All statistical tests were performed with Kyplotβ13 (Koichi Yoshioka) using a critical probability of *p*<0.05.

The fitting of concentration response curves were calculated according to y = {ymax−ymin/1+(x/EC_50_)n}+ymin (where ymax = response in the absence of agonist, ymin = response remaining in presence of maximal agonist concentration, x = concentration, EC_50_ = concentration of agonist producing 50% of the maximal response and n = slope) with Kaleidagraph 3.5 software (Synergy Software, Reading, PA, USA).

### Drugs

U73122, picrotoxin, CP55,940, THL and BAPTA from SIGMA (St. Quentin Fallavier, France) ; AM-251, DNQX, AM404, MPEP, and 2-amino-2-(2-carboxycyclopropan-1-yl)-3-(dibenzopyran-4-yl) propanoic acid (LY341495) from Tocris (Bristol, UK). URB 597 and URB 754 were from Cayman (SPI-BIO, Montigny Le Bretonneux, France). SR141716A was a generous gift from Sanofi-Aventis Recherche (Montpellier, France). Other chemicals were from the highest commercial grade available.

### Confocal microscopy

Mice (n = 4, 4–6 weeks old male, C57B1/6 strain) were deeply anesthetized with chloral hydrate (400 mg/kg body weight) and were transcardially perfused at room temperature (20–25°C) with PBS, pH 7.4, for 20 sec, followed by 500 ml of 4% formaldehyde (freshly depolymerized from paraformaldehyde) in 0.1 M phosphate buffer (PB), pH 7.4, for 10–15 min. Then, brains were removed from the skull and postfixed in 4% formaldehyde for up to 1 hr at room temperature. 50 µm-thick coronal sections cut from prPFC in a vibratome, were washed and blocked in 0.1 M PBS containing 3% newborn calf [25]primary antibodies for 2 days at 4°C. We used polyclonal CB1R goat antibodies (3 µg/ml in 1.5% NCS/PBS) and polyclonal DGL-α guinea pig and rabbit antibodies (2 µg/ml in 1.5% NCS/PBS) generously provided by Dr. Masahiko Watanabe (Department of Anatomy, Hokkaido University School of Medicine, Sapporo, Japan) [25], [26]. Polyclonal rabbit antibodies against mGluR5 (AB5675, Chemicon, CA, USA) were diluted at 1.36 µg/ml in 1.5% NCS/PBS. Slices were then washed in blocking solution and incubated with the following fluorochrome-conjugated secondary antibodies at working dilutions of 1:800 in 1.5% NCS/PBS: donkey anti-goat Alexa Fluor-488 (Molecular Probes, Eugene, OR, USA); donkey anti-rabbit Cy3 (Jackson ImmunoResearch Inc.); goat anti-guinea pig Alexa Fluor-594 and goat anti-rabbit Alexa Fluor-488 (Molecular Probes, Eugene, OR, USA). Incubations were overnight at 4°C. Slices were washed again in 0.1 M PBS and then mounted in Vectashield medium (Vector laboratories, Burlingame, USA), coverslipped, and imaged on a laser-scanning confocal microscope (Olympus Fluoview FV500). Photomicrographs were taken and presented using Adobe Photoshop 7 (Adobe Systems, San Jose, CA, USA).

### Double mGluR5/CB1R and DGL-α/CB1R immunocytochemistry for electron microscopy

Mice (n = 8, 4–6 weeks old male, C57B1/6 strain) were deeply anesthetized with chloral hydrate (400 mg/kg body weight). The animals were transcardially perfused with phosphate-buffered saline (PBS 0.1M, pH 7.4) and then fixed by 500 ml of a fixative made up of 0.1% glutaraldehyde, 4% formaldehyde (freshly depolymerized from paraformaldehyde) and 0.2% picric acid in PBS. Perfusates were used at 4°C.Tissue blocks were extensively rinsed in 0.1M PBS (pH 7.4). Coronal prPFC vibrosections were cut at 50 µm and collected in 0.1 M PBS (pH 7.4) at room temperature. Sections were preincubated in 10% blocking NCS serum prepared in PBS for 1 h at room temperature and then incubated overnight at room temperature with rabbit polyclonal antibodies to mGluR5 (AB5675, Chemicon, CA, USA; 1.36 µg/ml diluted in 1.5% NCS/PBS), or with polyclonal guinea pig antibodies to DGL-α (2 µg/ml in 1.5% NCS/PBS) generously gifted by Dr. Masahiko Watanabe (Department of Anatomy, Hokkaido University School of Medicine, Sapporo, Japan) for 2 days at 4°C. The localization of mGluR5 or DGL-α was carried out by means of a preembedding immunoperoxidase method. Briefly, prPFC sections were incubated sequentially at room temperature with a biotinylated secondary antibodies and avidin-biotin complex (ABC, Elite, Vector laboratories, Burlingame, CA, USA), each for 1 h. The immunoreaction was visualized with 0.05% 3,3′-diaminobenzidine (DAB)/ 0.01% hydrogen peroxide as chromogen.

The preembedding silver-intensified immunogold method described previously [27] was used for the localization of CB1R in prPFC sections processed for the immunoperoxidase method with either mGluR5 or DGL-α antiserum. Following incubation with the primary polyclonal goat antibodies against CB1R (3 µg/ml in 1.5% NCS/PBS; generously gifted by Dr. Masahiko Watanabe, Department of Anatomy, Hokkaido University School of Medicine, Sapporo, Japan) for 2 days, prPFC sections were incubated with 1.4 nm gold-labeled rabbit anti-goat IgG (Fab' fragment, 1:100, Nanoprobes Inc., Stony Brook, NY, USA) diluted in 1% NCS/PBS for 4 hours at room temperature. In another set of experiments, mGluR5/DGL-α and DGL-α/mGluR5 were colocalized by the preembedding immunogold and immunoperoxidase methods, being the first protein of each combination revealed by silver-intensified gold particles (mGluR5: 1.4 nm gold-labeled goat anti-rabbit IgG, Fab' fragment, 1:100; DGL-α: 1.4 nm gold-labeled goat anti-guinea pig IgG, Fab' fragment, 1:100; Nanoprobes Inc., Stony Brook, NY, USA). PrPFC tissue was subsequently postfixed in 1% glutaraldehyde for 10 min, rinsed extensively in double-distilled water, and gold particles were silver intensified with an HQ Silver kit (Nanoprobes Inc.) for about 8 min.

Successfully double stained sections were osmicated (1% OsO_4_ in 0.1 M PB, pH 7.4, 20 min), dehydrated in graded alcohols to propylene oxide and plastic-embedded flat in Epon 812. Ultrathin sections were collected on mesh nickel grids, stained with uranyl acetate and lead citrate, and examined in a JEOL (Peabody, MA) X-100 electron microscope.

Preparations were photographed by using standard electron microscopy negative plates. Figure compositions were scanned at 300 dots per inch (dpi). Labeling and minor adjustments in contrast and brightness were made with Adobe Photoshop (7.0, Adobe Systems, San Jose, CA, USA).

### Quantitative analysis

PrPFC sections from 3 mice were analyzed. Electron micrographs were taken at a final magnification of x15.000 from 150 µm-grid squares showing good and reproducible DAB immunoreaction and silver-intensified gold particles for any condition studied. Image-J (version 1.36) was used to measure the membrane length. Positive labeling was considered if immunoparticles were in close proximity to the plasmalemma. Metal particles on membranes and positive immunoreactive profiles were visualized and counted manually. The total area studied for CB1R was ≅340 µm^2^, mGluR5≅258 µm^2^ and DGL-α ≅306 µm^2^. Density of immunoparticles were averaged from different samples and presented as mean±SEM.
